# Polymers and Nanotechnology for Industry 4.0

**DOI:** 10.3390/polym15173556

**Published:** 2023-08-27

**Authors:** Ana M. Díez-Pascual

**Affiliations:** Universidad de Alcalá, Facultad de Ciencias, Departamento de Química Analítica, Química Física e Ingeniería Química, Ctra, Madrid-Barcelona Km. 33.6, Alcalá de Henares, 28805 Madrid, Spain; am.diez@uah.es

The term “polymer” derives from the Greek words “πολύς” meaning “many, much” and “μέρος” meaning “part”, and was proposed in 1833 by Jöns Jacob Berzelius, albeit with a different definition from the current IUPAC definition [1]. The modern concept of polymers, as covalently bonded macromolecular structures, was articulated in 1920 by Hermann Staudinger in his seminal work “Über Polymerisation” [2], in which he proposed that polymers were indeed long chains of atoms connected by covalent bonds. His work was questioned at length, but it was finally accepted by the scientific community. Owed to his work, Staudinger was awarded the Nobel Prize in 1953.

Despite the lack of theoretical knowledge, the potential of polymers to offer innovative, accessible and cheap materials was immediately claimed. The work of Braconnot, Parkes, Ludersdorf and many others on the modification of natural polymers determined many significant advances in the field [3]. Their contributions led to the discovery of materials such as celluloid, galalith, parkesine, rayon, vulcanised rubber and bakelite [4], all of which quickly entered industrial manufacturing processes and reached households as garments components (e.g., fabrics, buttons, decorative items and so forth). Currently there are innumerable uses of polymers in various industries due to their versatility and broad range of favourable characteristics that can be achieved through polymerisation, including electronic and photonic technologies, packaging and containers (films, bottles, food packaging, barrels, etc.), insulation, car parts (tires, bumpers, windshields, windscreen wipers, fuel tanks, car seats, etc.), medical applications, among many other possibilities [5,6,7,8].

This Special Issue compiles eight selected papers from the Proceedings of the 2nd International Online Conference on Polymers Science—Polymers and Nanotechnology for Industry 4.0 (IOCPS 2021) held on 1–15 November 2021 on sciforum.net, an online platform for hosting scholarly e-conferences and discussion groups. The conference was organised by the MDPI open access journal “Polymers” and provided researchers in the field of materials science and technology with the opportunity to present their research and exchange ideas with colleagues. 

The conference was organised into seven main sessions, providing a forum for presenting and discussing new results:

- Smart polymeric Synthesis and Modification for Industry 4.0;
- Polymer Development for Additive Manufacturing;
- Advanced Functional Testing of Polymeric Materials;
- Nanotechnologies in Polymer Science;
- Biotechnologies and Functional Biopolymers;
- Applications Polymers in the Industry 4.0;
- Polymer Recycling.

A free live streaming sessions was held on 12 November 2021 and included talks from invited speakers and a Q&A section to answer questions from a live online audience. The speakers were Prof. Dr. Ana María Díez-Pascual, with the presentation entitled “PEEK Composites with Carbon Nanomaterials”; Prof. Dr. Gianluca Cicala, with the talk “Hybrid Polymer-based Materials for Additive Manufacturing to Print Metal Parts”; and Prof. Miguel Aldas, with the speech “Revalorisation of Pine Resin Derivatives for the Development of Sustainable Polymeric Materials”.

The papers selected from the conference present widely varying types of polymers, such as polyurethane (PU) [9], cross-linked polyethylene (XLPE) [10], polycaprolactone (PCL) [11] and poly(L-lactide) (PLA) [11,12], microcapsules synthesized by the in situ polymerisation of crystal violet lactone (CVL), bisphenol A (BPA) and tetradecanol [13], polypropylene (PP) [14], and poly(3-hexylthiophene) [15]. In the following paragraphs, a concise overview of the published articles is provided in order to attract the interest of potential readers.

Polymers can be divided into natural and synthetic. Further, synthetic polymers can be classified into four groups: thermoplastics, thermosets, elastomers, and synthetic fibres. Thermoplastics can melt upon heating and generally have minimum cross-linking. They are more easily recyclable compared to thermosets and can withstand heating and reforming processes. Amongst the most used thermoplastics are polyethylene (PE) and polypropylene (PP) [16]. PE is frequently used for manufacturing zip lock bags, bottles, food containers, food crates, stretch film, tape, pallets and plastic sheeting (Figure 1). 

Another application of XLPE is in the field of high voltage direct current (HVDC) technology [17], in particular as cable insulation material. In this regard, Hang et al. [10] have investigated the physical, thermal, mechanical, and space charge properties of commercial AC and DC XLPE cable materials. Thermal analysis experiments and oven aging tests were performed to assess their oxidation and thermal-oxidative aging. Further, the crosslinking byproducts, as well as their antioxidant content and oxidation resistance were evaluated. XPS analysis revealed that the XLPE samples treated at 250 °C still comprised residual crosslinking byproducts. These byproducts were a crucial factor that led to the deterioration of the electrical properties, especially space charge accumulation and internal electric field distortion. 

Polypropylene fabric is a textile currently used for upholstery, industrial and manufacturing applications (Figure 1) as it is soft, lightfast, and easy to clean since it has no active dye sites. The production of PP at an industrial scale comprises several stages, namely the initiation reaction where the activation occurs; the polymerisation stage, in which the chain of monomers is formed; and finally the termination stage, in which the chain formation reaction is terminated [18]. In this process, a catalyst (Ziegler–Natta (ZN) catalyst), a co-catalyst (triethyl aluminium (TEAL)), and gases (hydrogen and nitrogen) are used. Sulphur compounds are removed from propylene via purification processes. Nevertheless, these processes are not completely effective; consequently low concentrations of compounds such as H_2_S are are present in commercial PP. Hernandez-Fernandez [14] investigated the effects of H_2_S content on PP polymerisation through the controlled dosage of this compound with concentrations between 0.07 and 5 ppm to determine possible reaction mechanisms. Further, they assessed the change in the material properties via a variety of technique, such as TGA, FTIR, MFI and XDR analysis. Traces of various impurities can be found after the synthesis process, thereby reducing the polymerisation yield and producing materials that must undergo revaluation processes such as pyrolysis.

On the other hand, thermosets have a high degree of crosslinking, which makes them insoluble and non-melting when heated. Due to their difficult recyclability, they are not environmentally friendly. They can be moulded when heated and solidified when cooled. Thermosets are typically resins or synthetic plastics that harden permanently under heat and pressure. Amongst the most common thermosets are polyurethanes (PUs), which are utilised as adhesives, sealants, and coatings in building and infrastructures (Figure 1). Further, they are widely used as matrices in polymeric composites. PUs are prepared via polyaddition reaction, involving isocyanates and compounds containing hydrogen-donating groups, primarily hydroxyls. The reaction between isocyanates and hydroxyl groups yields the urethane group. Stoichiometric changes between these groups condition the occurrence of additional reactions, especially reactions of isocyanates. In this regard, the structure and performance of PU/ground tire rubber (GTR) composite foams with different isocyanate indexes (from 0.8 to 1.2) have been investigated [9]. Integrating GTR into the PU matrix reduced the average cell diameter compared to the unfilled foams. Further, a shift in the glass transition temperature was found, which enhanced the composite stiffness, while the thermal stability was reduced.

Conductive polymers are organic polymers that conduct electricity. They are easy to process, primarily by dispersion. Usually, they are not thermoplastics, i.e., they are not thermoformable. They can offer high electrical conductivity but do not show similar mechanical properties to other commercially available polymers. The electrical properties can be fine-tuned using organic synthesis methods and by advanced dispersion techniques. Poly(3-hexylthiophene) (P3HT) is a regioregular conductive polymer. It is a p-type semiconductor and is one of the most attractive polymers as donor material in organic solar cells due to its outstanding chemical and electrochemical stability, high electrical conductivity, and an appropriate HOMO energy level of approximately −4.9 eV [19]. Moreover, P3HT has a high absorption coefficient and can absorb more than 95% of the solar spectrum over a wavelength range of 450–600 nm, which makes it extremely attractive for organic optoelectronic devices [20]. Recently, a review of the changes in electrical conductivity, bandgap, hole collection properties and carrier mobility of P3HT when adding graphene (G) has been reported [15]. The main objective was to assess how the addition of different G loading influences the optical constants: refractive index (n) and extinction coefficient (k). The values of n and k for six P3HT/G nanocomposites with G concentrations between 0.1 and 5 wt% were fitted to two different models, Forouhi Bloomer and Cauchy, showing very good agreement between the experimental and theoretical values. 

Polycaprolactone (PCL) is a biodegradable, semi-crystalline thermoplastic polyester produced by cationic or anionic ring-opening polymerisation of ε-caprolactone at elevated temperatures (≤200 °C) and in the presence of a suitable catalyst. Polylactic acid (PLA) is biodegradable hydrolysable aliphatic semicrystalline polyester produced through the direct condensation reaction of its monomer. It is widely used in biomedical applications, such as sutures, stents, dialysis media, and drug delivery devices. It is also being evaluated as a material for tissue engineering; loose-fill packaging, compost bags, food packaging, and disposable tableware. New block copolymers of methyl ethylene phosphate (MeOEP) with caprolactone (CL) and L-lactide (LA) were examined via Raman spectroscopy [11]. An analysis of the peak positions, relative intensities and profiles of the poly(methyl ethylene phosphate) (PMeOEP), PCL and PLA bands showed that the conformational and phase compositions of each constituent in the copolymers did not differ significantly from those of the homopolymers. Data on the conformational and phase copolymer composition are valuable for the prediction and explanation of their physical and chemical properties. 

A novel method to improve the properties of textile materials is the incorporation of natural ingredients into the textile products during the manufacturing process. Bolskis et al. [12] investigated the formation of biodegradable melt-spun multifilaments of PLA with different contents (5%, 10% and 15%) of a natural material (pine rosin). Nanocomposites were prepared by melt-spinning at two different draw ratios (1.75 and 2.75). The former draw ratio caused the formation of brittle PLA and PLA/rosin yarns. The presence of rosin reduced the mechanical properties of the melt-spun PLA/rosin multifilament, namely tenacity, tensile strain and elongation at break, while the absorbance in the whole UV region increased. In addition, the melting point and degree of crystallinity decreased whilst the wetting angle increased compared with pure PLA multifilament. Overall, biodegradable nanocomposites from removable resources show great potential as an alternative to petroleum-based polymeric materials and are expected to be the focus of intensive research in the near future.

## Figures and Tables

**Figure 1:**
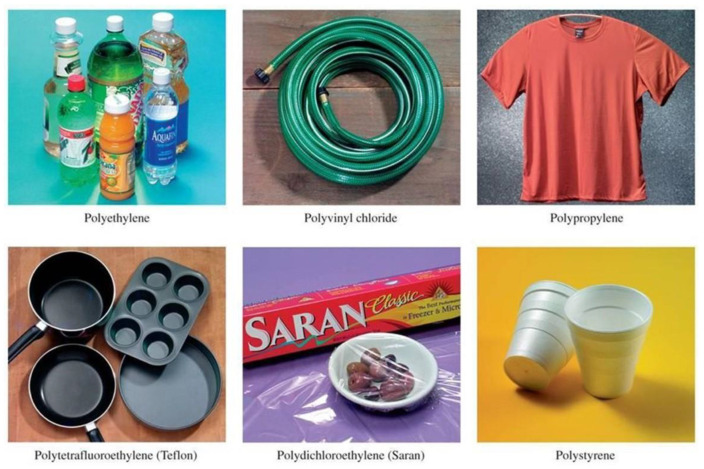
Representative examples of applications of synthetic polymers in everyday life.
